# Magnetic and Electronic Properties of Weyl Semimetal Co_2_MnGa Thin Films

**DOI:** 10.3390/nano11010251

**Published:** 2021-01-19

**Authors:** Peter Swekis, Aleksandr S. Sukhanov, Yi-Cheng Chen, Andrei Gloskovskii, Gerhard H. Fecher, Ioannis Panagiotopoulos, Jörg Sichelschmidt, Victor Ukleev, Anton Devishvili, Alexei Vorobiev, Dmytro S. Inosov, Sebastian T. B. Goennenwein, Claudia Felser, Anastasios Markou

**Affiliations:** 1 Max Planck Institute for Chemical Physics of Solids, Nöthnitzer Str. 40, 01187 Dresden, Germany; Peter.Swekis@cpfs.mpg.de (P.S.); Alexandr.Sukhanov@cpfs.mpg.de (A.S.S.); ycchen.mse00@g2.nctu.edu.tw (Y.-C.C.); Gerhard.Fecher@cpfs.mpg.de (G.H.F.); Joerg.Sichelschmidt@cpfs.mpg.de (J.S.); Claudia.Felser@cpfs.mpg.de (C.F.); 2 Institut für Festkörper- und Materialphysik, Technische Universität Dresden, 01062 Dresden, Germany; dmytro.inosov@tu-dresden.de (D.S.I.); Sebastian.Goennenwein@tu-dresden.de (S.T.B.G.); 3 Department of Materials Science and Engineering, National Chiao Tung University, Hsinchu 30010, Taiwan; 4 Deutsches Elektronen-Synchrotron DESY, 22607 Hamburg, Germany; andrei.hloskovsky@desy.de; 5 Department of Materials Science and Engineering, University of Ioannina, 45110 Ioannina, Greece; ipanagio@uoi.gr; 6 Laboratory for Neutron Scattering and Imaging, Paul Scherrer Institute, 5232 Villigen, Switzerland; victor.ukleev@psi.ch; 7 Institut Laue Langevin, 38000 Grenoble, France; devishvili@ill.eu; 8 Department of Physics and Astronomy, Uppsala University, 75120 Uppsala, Sweden; Alexey.Vorobiev@physics.uu.se; 9 Center for Transport and Devices of Emergent Materials, Technische Universität Dresden, 01062 Dresden, Germany; 10 Department of Physics, University of Konstanz, 78457 Konstanz, Germany

**Keywords:** topological materials, magnetic Weyl semimetals, Heusler compounds, magnetic dichroism, photoelectron spectroscopy, ferromagnetic resonance, polarized neutron reflectivity, thin films, magnetic anisotropy

## Abstract

Magnetic Weyl semimetals are newly discovered quantum materials with the potential for use in spintronic applications. Of particular interest is the cubic Heusler compound Co_2_MnGa due to its inherent magnetic and topological properties. This work presents the structural, magnetic and electronic properties of magnetron co-sputtered Co_2_MnGa thin films, with thicknesses ranging from 10 to 80 nm. Polarized neutron reflectometry confirmed a uniform magnetization through the films. Hard x-ray photoelectron spectroscopy revealed a high degree of spin polarization and localized (itinerant) character of the Mn *d* (Co *d*) valence electrons and accompanying magnetic moments. Further, broadband and field orientation-dependent ferromagnetic resonance measurements indicated a relation between the thickness-dependent structural and magnetic properties. The increase of the tensile strain-induced tetragonal distortion in the thinner films was reflected in an increase of the cubic anisotropy term and a decrease of the perpendicular uniaxial term. The lattice distortion led to a reduction of the Gilbert damping parameter and the thickness-dependent film quality affected the inhomogeneous linewidth broadening. These experimental findings will enrich the understanding of the electronic and magnetic properties of magnetic Weyl semimetal thin films.

## 1. Introduction

Topological materials have attracted tremendous interest in condensed matter physics due to their unique electronic band states, which give rise to novel linear and nonlinear responses [1,2,3,4,5,6,7,8,9]. Weyl semimetals constitute one class of such topological systems. They are characterized by a lack of inversion symmetry or broken time-reversal symmetry, resulting in two-fold degenerate band-touching points (Weyl nodes) with opposite chirality formed in momentum space [3,5,10,11,12]. The Weyl nodes act as quantized sources and sinks of Berry curvature, which in turn lead to intriguing transport properties, such as the anomalous Hall effect [13,14,15,16,17,18], the anomalous Nernst effect [19,20,21,22], magneto-optical responses [23] and the chiral anomaly [24,25]. Recently, some ferromagnetic compounds were proposed to be time reversal symmetry breaking Weyl semimetals (WSMs). Examples of such materials are Heusler compounds [26,27,28] and kagome crystals [29,30,31].

In particular, the Heusler compound Co_2_MnGa has received significant attention since the recent experimental verification of Weyl fermion lines and drumhead topological surface states [32]. Co_2_MnGa crystallizes in the Cu_2_MnAl-type structure (*L*2_1_, space group $Fm\bar{3}m$, #225) with a Curie temperature of *T*_C_ = 694 K and saturation moment of *M*_s_ = 4.05 *µ*_B_ per formula unit [33]. Single crystals and thin films exhibit Berry curvature-driven large anomalous Hall and Nernst effects [16,34,35,36,37,38,39]. Further, Co_2_MnGa films show negative magnetoresistances [40] and high spin polarization [41,42], which in turn results in low magnetic damping [42,43]. Interestingly, Pechan et al. reported that Co_2_MnGa films grown on different seed layers achieved tunable strain states, which induced remarkably large two-fold and four-fold in-plane (IP) anisotropies [44].

Despite the promising properties of Co_2_MnGa, knowledge about the intrinsic magnetic properties of thin films in the chemically ordered *L*2_1_ structure remains limited. Thus, to fully utilize Co_2_MnGa in practical applications, e.g., spintronic and magnetic memory devices, it is necessary to understand its electronic and magnetic properties, particularly the dynamic magnetic properties approaching the thin film limit. Key parameters include the Gilbert damping, saturation magnetization and magnetic anisotropies.

In this work, we systematically studied the structural, electronic and magnetic properties of high-quality heteroepitaxial *L*2_1_-ordered Co_2_MnGa thin films grown on MgO(001) single crystal substrates, with thicknesses ranging from 10 to 80 nm. We obtained a uniform depth profile of the film magnetization using polarized neutron reflectometry (PNR). Further, we investigated the 2*p* core levels of Co and Mn by means of magnetic dichroism in hard x-ray photoelectron spectroscopy (HAXPES) to infer the itinerant and localized characters of the respective *d* valence electrons and accompanying magnetic moments. In addition, we related the thickness-dependent structural properties to the magnetic properties, including the inhomogeneous linewidth broadening, Gilbert damping parameter and magnetic anisotropies, as determined by ferromagnetic resonance (FMR) experiments. In that context, we found that the films showed cubic anisotropy within the film plane and uniaxial anisotropy perpendicular to the film plane.

## 2. Materials and Methods

High-quality epitaxial thin films of Co_2_MnGa were grown in a BESTEC UHV magnetron sputtering system on single crystal MgO(001) substrates and capped with 3 nm Al, which is naturally oxidized and protects the epilayer. The details of the growth are provided in [34]. The stoichiometry of the films was confirmed by energy-dispersive X-ray spectroscopy (EDXS), with an experimental uncertainty of less than 5 at. %. X-ray diffraction (XRD) and X-ray reflectivity (XRR) measurements were conducted using a PANalytical X’Pert^3^ MRD diffractometer employing Cu-Kα_1_ radiation (λ = 1.5406 Å). The film thicknesses were determined by using XRR measurements (not shown). Atomic force microscopy (AFM) images were collected in non-contact mode on an MFP-3D Origin^+^ microscope from Oxford Instruments Asylum Research in replicas of the films without capping layers.

PNR measurements were conducted on the SuperADAM instrument at ILL (Grenoble, France). A fixed neutron wavelength of 5.2 Å with an incident polarization of 99.6% was used for the measurements. The neutron wavelength spread was *δλ/λ* ≈ 0.5%. The neutron momentum transfer was selected by changing the incident and outgoing angles to satisfy the specular reflection condition. In addition, the incident neutron polarization state was controlled by a radiofrequency spin flipper for each of the consecutive measurements. The data were collected at an applied magnetic field of 50 mT preceded by the application of a 0.7 T field. For the details of the SuperADAM polarized neutron reflectometer, see [45,46].

The HAXPES measurements were performed at beamline P22 of PETRA III (Hamburg, Germany) [47]. The photon energy was set to *hν* = 6000 ± 0.1 eV. The magnetic circular dichroism was measured at a fixed magnetization by changing the helicity of the photons using a phase retarder. The degree of circular polarization was about 98–99%. The thin films were magnetized in situ along the direction of the photon beam. The energy in the spectra is given with respect to the Fermi energy *ε*_F_ calibrated to Au, with *ε*_F_ appearing at a kinetic energy *E*_kin_ of 6000.50 ± 0.2 eV. This corresponds to an overall energy resolution of about 170 meV (*E*/Δ*E* ≈ 3.5 × 10^4^). For details of the HAXPES setup and HAXPES-MCDAD experiment, see [48,49].

Magnetization measurements were performed on a Superconducting Quantum Interference Device (SQUID) vibrating sample magnetometer (MPMS 3, Quantum Design). To infer the magnetic field-dependent magnetization of the films, we subtracted the diamagnetic substrate contribution from the raw data.

Broadband FMR measurements were performed on a coplanar waveguide (CPW) in a vector network analyzer (VNA) setup. The CPW was connected to two ports of the VNA, and the complex scattering parameter S_21_ was recorded by sweeping the frequency with a constant field applied perpendicular to the film plane. Consecutive measurements were performed in 0.5 mT steps. The resonance field *H*_res_ and linewidth Δ*H* were extracted by fitting the field dependence of the complex transmission at constant frequency S_21_(*H*)|_f_ to the complex Polder susceptibility, as discussed by Nembach et al. [50].

FMR measurements as a function of the external magnetic field orientation were performed on a continuous-wave Elexsys E500 spectrometer by Bruker. The measurements were conducted at X-band microwave frequencies (*ω* = 9.4 GHz) in a cylindrical cavity (TE_011_ mode). The resonance signal was recorded in the field-derivative d*P*/d*H* of the absorbed microwave power (*P*) using a lock-in technique that modulated the external field at a low frequency (100 kHz). The sample orientation was manipulated by a goniometer, rotating perpendicular to *H*. The obtained spectra were fitted with a first-derivative Lorentzian line shape to obtain the resonance field *H*_res_.

## 3. Results

### 3.1. Structural and Morphological Characterization

**Figure 1:**
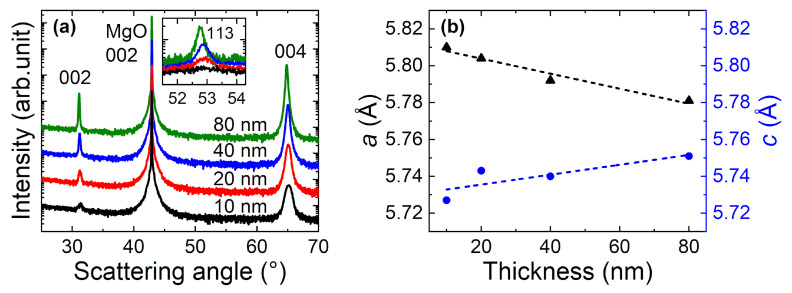
(**a**) XRD patterns of Co_2_MnGa films with different thicknesses. The inset shows the asymmetric 113 reflections. (**b**) Lattice parameters of Co_2_MnGa films as a function of thickness.

Figure 1a shows the symmetric radial *ω*–2*θ* XRD patterns of Co_2_MnGa films with different thicknesses. We observe only the 00*l* reflections of Co_2_MnGa, which suggest that the films grow heteroepitaxially on the MgO(001) substrates. The inset in Figure 1a portrays the asymmetric 113 superstructure reflections. From those, we determined that all of the films crystallized with *L*2_1_-type chemical ordering [34]. By combining the symmetric and asymmetric reflections, such as 002 and 220, respectively, from XRD measurements, we estimated the lattice parameters of our films, which are depicted in Figure 1b. The thinner films show strain-induced tetragonal distortion, whereas the thicker films are closer to the cubic bulk value (*a* = 5.77 Å) [33,34]. The films are under bi-axial tensile strain as expected based on the difference between the lattice parameters of the films and substrate ($\sqrt{2}$*a*_MgO_ = 5.956 Å).

The misfit strain (averaged through the film thickness) increases from 2.51% at 80 nm to 3.63% at 10 nm. Table 1 summarizes the structural parameters.

**Figure 2:**
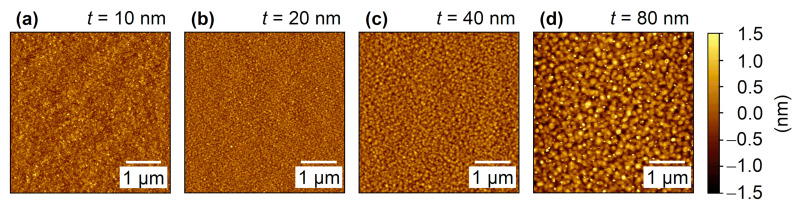
(**a**–**d**) AFM topographic images of the Co_2_MnGa films with different thicknesses.

Figure 2 illustrates the AFM topographic images for the Co_2_MnGa films with different thicknesses in an area of 5 × 5 μm^2^. The films exhibit smooth surfaces, with an average RMS roughness *S*_q_ that increases from 2.56 to 5.44 Å with increasing film thickness. Simultaneously, the mean lateral surface diameter of the grains *D* increases with increasing film thickness. The roughness is expected to be proportional to the grain size. However, according to the results shown in Table 1, the minimum roughness is obtained for the 20 nm sample. This is not totally unexpected, as, in the initial stages (10 nm) of growth of a metallic film on an insulating substrate, island formation normally occurs. Simultaneously, the morphology is defined by kinetic mechanisms. At elevated substrate temperatures (550 °C), accelerated recrystallization and grain growth result from rapid surface diffusion coupled with mobile dislocations and grain boundaries. Further, the improved crystallinity inferred from rocking curve measurements [34] indicates that the overall film quality increases with increasing film thickness. Similar behavior can also be observed in other thin films [51,52]. Table 1 summarizes the values of *S*_q_ and *D*.

### 3.2. PNR

**Figure 3:**
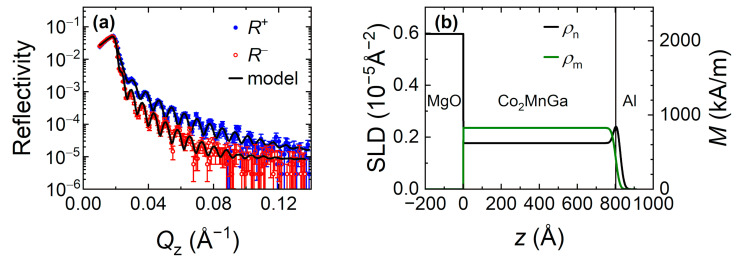
(**a**) PNR measurements of an 80-nm-thick Co_2_MnGa film. The solid lines are the fitted curves. (**b**) Nuclear and magnetic SLD profiles obtained from fitting the PNR data. The right axis shows the magnetization corresponding to the magnetic SLD profile.

To determine the magnetic depth profile of an 80-nm-thick Co_2_MnGa film, we performed PNR measurements. Figure 3a shows the intensity of the specular reflection for two incident neutron polarizations: *R*^+^ (spin up, along the applied field) and *R*^−^ (spin down) as a function of the momentum transfer perpendicular to the film surface (*Q*_z_). The data exhibit well-defined oscillations that are resolved up to ~0.14 Å^−1^. Reflectivity measurements for the two neutron polarizations enabled us to separate the contributions from the nuclear (*ρ*_n_) and magnetic (*ρ*_m_) scattering length density (SLD) profiles. To extract the SLD profiles, we fitted the experimental data with a model that included the Al capping layer and magnetic Co_2_MnGa layer on top of the MgO substrate using the GenX software [53]. To achieve the most accurate refinement of the magnetic profile, the nuclear SLD values for the MgO substrate and Co_2_MnGa layer were fixed to the values calculated based on the lattice constants. Thus, the fitted parameters included the thicknesses of the Co_2_MnGa and capping layers, roughness of each interface and magnetization of the Co_2_MnGa layer. The applied model resulted in good agreement with the experimental data, as demonstrated by the fitting curves in Figure 3a.

Figure 3b presents the corresponding SLD profiles. The interface between the substrate and Co_2_MnGa layer is relatively sharp (the roughness is less than the measurement sensitivity). The Co_2_MnGa layer has a refined thickness of 80.3 nm and exhibits a roughness of 1.7 nm at the interface with the capping layer. The SLD of the 1.1-nm-thick capping layer is slightly higher than the theoretical SLD of Al (~0.21 × 10^−5^ Å^−2^), which indicates the presence of an oxide layer (Al_2_O_3_ has a higher SLD) at the film surface. As shown by the good quality of the fit, the model that assumes a uniform magnetization within the Co_2_MnGa layer well reproduces the experimental data. The magnetization calculated from the magnetic SLD [54] is 827 ± 49 kA/m.

### 3.3. HAXPES

**Figure 4:**
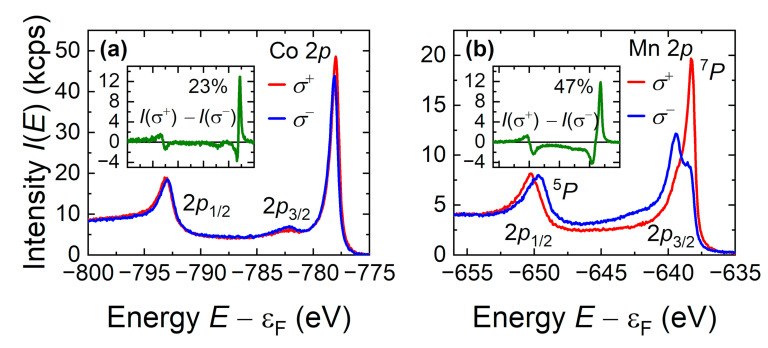
(**a**) Co 2*p* and (**b**) Mn 2*p* HAXPES spectra of Co_2_MnGa film on MgO(001). Shown are spectra taken with *σ*^+^ and *σ*^−^ polarization of the photons. The insets show the difference spectra, that is the dichroism.

Figure 4 depicts the polarization-dependent core-level spectra near the Co and Mn 2*p* excitations for a 40-nm-thick Co_2_MnGa film. Note that the spectra were taken from remanently magnetized samples; hence, the magnetic moment may be lower than the saturation moment. The dichroism is quantified by an asymmetry defined as:

(1) $$A=\frac{I^{+}-I^{-}}{\max\left(I_{0}-I_{bg}\right)}$$

where $I^{+}$ and $I^{-}$ are the intensities for opposite helicities, $I^{+}-I^{-}=I_{CD}$ is the dichroism, $I_{0}=I^{+}+I^{-}$ is the sum of the intensities and $I_{bg}$ is the background intensity.

Figure 4a shows the polarization-dependent spectra and dichroism in the energy region of the Co 2*p* states. The Co 2*p* state exhibits a spin–orbit splitting of Δ_SO_ = 15 eV into the 2*p*_1/2_ and 2*p*_3/2_ sub-states, slightly larger than that of Co_2_MnSi [49]. The dichroism exhibits a sign change (+ −−+) across the energy range of the 2p excitation, which is typical for Zeeman-type level ordering in the single-electron model [55]. A pronounced satellite is observed at about 4.3 eV below the 2*p*_3/2_ state but is not detectable at the 2*p*_1/2_ state. Further, the 2*p*_3/2_ excitation exhibits a splitting of about 100 meV. The asymmetry (Equation (1)) varies between +23% and –6% across 2*p*_3/2_ and between –6% and +5% across 2*p*_1/2_. Both the polarization-dependent spectra and dichroism indicate that the lines of the multiplet extend over the entire spectral range. In particular, the dichroism does not vanish between the two main parts of the spin–orbit doublet. Comparison with calculated spectra [49] revealed a *jj*-type coupling in accordance with multiplet calculations [56]. The Zeeman-type splitting observed at both lines is caused by the exchange interaction. The dichroism at the Co 2*p* states is close to that observed for exchange-biased CoFe or Co_2_FeAl films [57].

The polarization-dependent 2*p* spectra of Mn in Figure 4b exhibit a more complicated structure. Splittings of Δ_1/2_ ≈ 1 eV and Δ_3/2_ ≈ 1.3 eV occur at the 2*p*_1/2_ and 2*p*_3/2_ excitations, respectively. The total intensity $I_{0}$ (not shown) does not reveal spin-orbit splitting due to the additional splitting of both lines, 2*p*_1/2_ and 2*p*_3/2_. The mean splitting between the doublet-type structure amounts to about Δ = 11 eV, similar to that in Co_2_MnSi [49]. In atoms, multiplet splitting occurs due to the interaction of the *nl*^−1^ core hole with the polarized open valence shell. The core hole (here 2*p*^5^) in a solid is expected to interact with the polarized *d* states of the valence band. The localized valence *d* states, however, are screened by delocalized electrons; therefore, quantification is not easily possible. The multiplet theory can be used to explain the observed splittings in the spectra [49,58,59,60], assuming that the atomic character of the valence electrons is partially retained in the solid. Comparison indicated that the two parts of the multiplet could be assigned to the ^5^*P* and ^7^*P* states with the dipole allowed transitions $\left\{\left[2p^{5}3d^{5}{(}^{5,7}P_{j}\right]+\epsilon \left(s,d\right)\right\}{(}^{6}P_{7/2,5/2,3/2})$. The dichroic asymmetry across the 2*p*_3/2_-type part varies between +47% and −24% and does not vanish between the 2*p*_3/2_ and 2*p*_1/2_ lines. Thus, the splitting is not of Zeeman type, where no additional states would appear between the main lines of the spin–orbit doublet similar to the Co 2*p* state. Hence, the core hole created by the emission of a Mn 2*p* electron interacts strongly with the valence band. The multiplet structure and magnetic dichroism of the Mn 2*p* states of Co_2_MnGa are very similar to those of Co_2_MnSi [49], indicating the similarity of the electronic structures, in particular, half-metallic character with very high spin polarization.

### 3.4. Static and Dynamic Magnetic Properties

#### 3.4.1. DC Magnetometry

**Figure 5:**
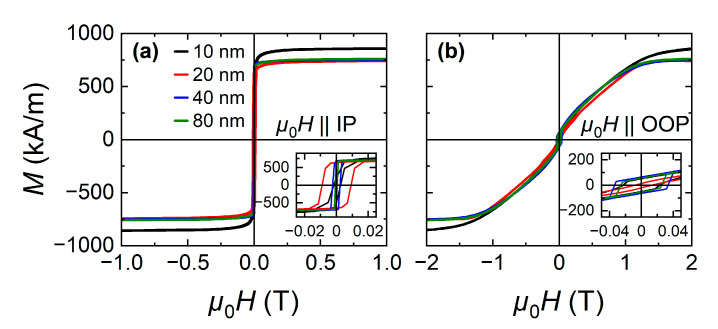
Magnetization hysteresis loops with *H* applied along the: (**a**) IP and (**b**) OOP film directions at 300 K for Co_2_MnGa films of various thicknesses.

Figure 5 depicts the IP and out-of-plane (OOP) magnetization hysteresis loops at 300 K for the Co_2_MnGa films of various thicknesses. All films show magnetization hysteresis loops characteristic for soft ferromagnetic materials with high magnetization and small coercivity. Magnetic saturation (*M*_s_) is reached easily along the IP direction, which is the easy magnetic axis, while the hard magnetic axis is normal to the film plane along the OOP direction. Between 80 and 20 nm the *M*_s_ is similar, while for the thinner film of 10 nm we observe the highest *M*_s_. This enhanced *M*_s_ can be attributed to the strain-induced changes of the electronic structure.

#### 3.4.2. Broadband FMR

**Figure 6:**
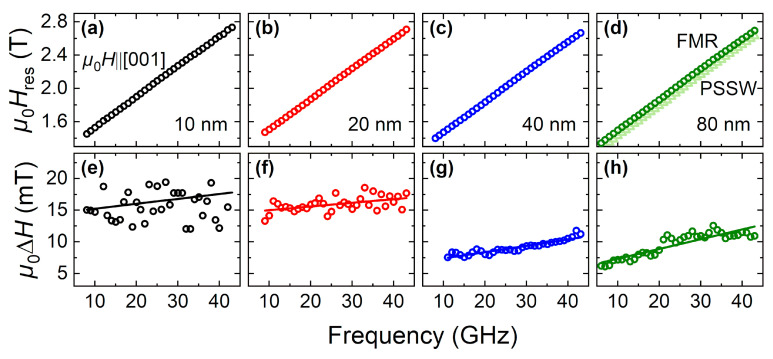
(**a**–**d**) Resonance fields *µ*_0_*H*_res_ with *H* applied perpendicular to the film plane for the Co_2_MnGa films with various thicknesses; and (**e**–**h**) linewidths of the respective FMR modes, including linear fits for the damping parameters.

We performed broadband FMR measurements of the Co_2_MnGa films at 300 K with *H* applied perpendicular to the film plane. This configuration ensured minimization of the two-magnon scattering contribution to the extrinsic broadening of the linewidth [61]. Figure 6a–d presents *H*_res_ of the FMR mode as a function of the excitation frequency for the Co_2_MnGa films. Notably, for the 80-nm-thick film (Figure 6d), *H*_res_ of another mode in addition to the uniform FMR mode was extracted at lower fields. Aside from possible film non-uniformities, the origin of this mode could be a perpendicular standing spin wave (PSSW) with a nonzero wave vector q=np/t (integer order of mode *n*, with *n* = 0 as the uniform mode) pointing perpendicular to the film plane. Here, the absence of additional higher order modes may be related to a very weak excitation, resulting in an intensity below the detection limit and preventing us from attributing this mode to a PSSW with certainty.

To determine the effective magnetization *M*_eff_, *g*-factor and exchange stiffness *A*, a simplified resonance condition was evaluated. For parallel *M* and *H* (applied perpendicular to the film surface), this gives:

(2) $$\frac{\omega }{\gamma }=\mu_{0}\;\left(H_{\mathrm{res}}-M_{\mathrm{eff}}+\frac{2An^{2}\pi^{2}}{M_{s}t^{2}}\right)$$

with the gyromagnetic ratio $\gamma =\frac{g\mu_{B}}{\hbar }$. The exchange stiffness was only extracted for the 80-nm-thick film, assuming that the second mode originated from a PSSW. Here, *A* = 16.8 pJ/m is comparable to the exchange stiffness in other Co-based Heusler compounds (4.8–31.5 pJ/m) [62,63,64]. Table 2 summarizes *M*_eff_ and *g* for all of the investigated Co_2_MnGa films. As the thickness increases, *M*_eff_ drastically decreases. Further, a noticeable uniaxial magnetic anisotropy perpendicular to the film plane, attributed to the growth-induced lattice strain, can be inferred from the increased size of *M*_eff_ compared to *M*_s_.

Figure 6e–h presents Δ*H* as a function of the excitation frequency for the Co_2_MnGa films. Notably, the data for the 10-nm-thick film show significant scattering, attributed to a less accurate fit of the FMR mode due to the low signal intensity. Δ*H* characterizes the relaxation of the magnetization due to extrinsic frequency-independent contributions (inhomogeneous linewidth broadening Δ*H*_0_) as well as intrinsic contributions linearly proportional to the resonance frequency (Gilbert damping parameter α). These contributions can be determined from the frequency dependence of Δ*H* as:

(3) $$\mu_{0}\Delta H=\mu_{0}\Delta H_{0}+\frac{2\omega }{\gamma }$$

The α values summarized in Table 2 agree well with previously reported values for Co_2_MnGa films (α ≈ 2 × 10^−3^) [42,43]. In that context, the low α values in conjunction with the previously inferred high degree of spin polarization agree well with the correlation of those properties established by Liu et al. [65]. Further, α slightly increases with thickness. This thickness dependence likely originates in the lattice distortion affecting the spin polarization [66]. In contrast, Δ*H*_0_ decreases with increasing film thickness, which indicates increased inhomogeneities in the thinner films and lower crystalline quality [34].

#### 3.4.3. X-Band FMR

**Figure 7:**
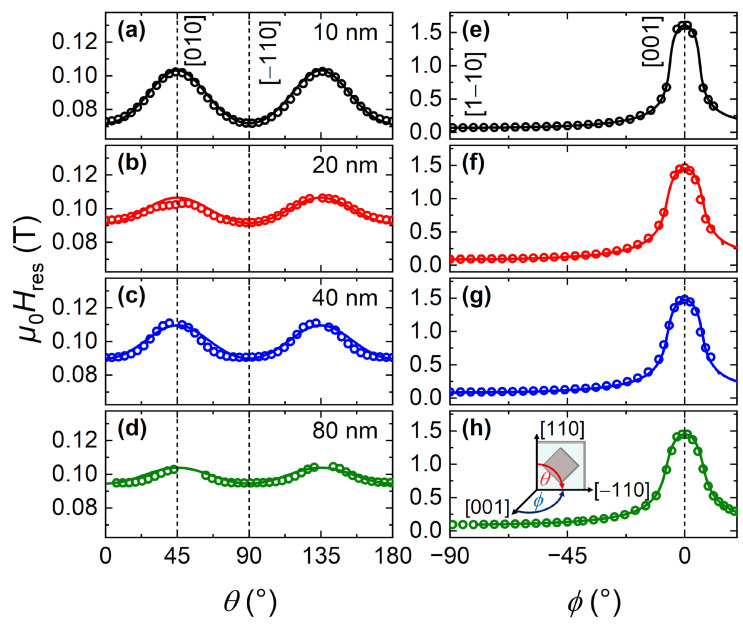
Resonance fields *H*_res_ and simulated resonance conditions (solid lines, see main text) at 300 K and 9.4 GHz of Co_2_MnGa films with different thicknesses: (**a**–**d**) *H* rotated in the (001) plane, i.e., IP rotation; and (**e**–**h**) *H* rotated in the (110) plane, i.e., IP to OOP rotation. The dashed lines indicate the crystallographic directions of the film at the respective angles. Inset in (**h**): Cartesian and polar coordinate system, where the crystallographic directions refer to the Co_2_MnGa film and the angles to the direction of *H*.

Figure 7 shows the *H* orientation dependencies of *H*_res_ in the (001) plane (IP) and (110) plane (IP to OOP). Note that, for the 80-nm-thick film, we only considered the main mode. In the (001) plane (Figure 7a–d), *H*_res_ has a four-fold *H* orientation dependence, which can be explained by the cubic symmetry of the Heusler structure. Here, the easy axes lie along the [110] and equivalent directions. In the (110) plane (Figure 7e–h), the *H* orientation dependence of *H*_res_ agrees with the magnetization measurements (cf. Figure 5), with the magnetic hard axis along the (001) axis (OOP).

The magnetic anisotropies of the Co_2_MnGa films can be determined from the dependence of the FMR resonance condition on the direction of the applied magnetic field *H* with respect to the growth orientation of the crystallographic axes (inset in Figure 7h). For that purpose, the total free energy density (*F*_tot_) was employed. Here, we describe *F*_tot_ of the Co_2_MnGa films using the Zeeman energy, the shape anisotropy, a uniaxial anisotropy term perpendicular to the film plane and a cubic anisotropy term:

(4) $$\begin{array}{ll} F_{tot}= & -\mu_{0}M_{s}H\left(\sin\Theta \sin\Phi \sin\theta \sin\phi +\cos\Theta \cos\theta +\sin\Theta \cos\Phi \sin\theta \cos\phi \right)\\ & +\frac{\mu_{0}}{2}M_{s}^{2}\sin^{2}\Theta cos^{2}\Phi \\ & -K_{u,\left[001\right]}{\left(\sin\Theta cos\Phi \right)}^{2}\\ & +K_{c}\frac{1}{4}\left(\sin^{2}\left(2\Theta \right)+\sin^{4}\Theta \sin^{2}\left(2\Phi \right)\right). \end{array}$$

(Θ,Φ) and (θ,ϕ) correspond to the angles of the magnetization and applied magnetic field defined in relation to the sample, respectively. *K*_u,[001]_ and *K*_c_ are the perpendicular uniaxial and cubic anisotropy constants, respectively. The resonance condition can, in turn, be derived from *F*_tot_ for arbitrary orientations of the external magnetic field with respect to the sample as [67]:

(5) $${\left(\frac{\omega }{\gamma }\right)}^{2}=\frac{1}{M_{s}^{2}\sin\Theta }\left(\frac{\partial^{2}F_{\mathrm{tot}}}{\partial \Phi^{2}}\frac{\partial^{2}F_{\mathrm{tot}}}{\partial \Theta^{2}}-{\left(\frac{\partial^{2}F_{\mathrm{tot}}}{\partial \Theta \partial \Phi }\right)}^{2}\right)$$

with the derivatives evaluated for the equilibrium direction of the magnetization. In addition, for the simulation of the resonance condition in the presented coordinate system, ϕ was fixed at 90° for rotation in the film plane, whereas θ was fixed at 90° for rotation out of the film plane.

From the *H*_res_ simulation results (Figure 7, solid lines) obtained using Equations (4) and (5), the anisotropy constants at 300 K were derived, using *M*_s_ and *g* (entering via $\gamma =\frac{g\mu_{B}}{\hbar }$) in Table 2. The simulations well reproduced both rotation planes, and Table 3 summarizes the corresponding anisotropy constants.

Based on the previous *M*_eff_ results (cf. Table 2), the Co_2_MnGa films indeed show a uniaxial contribution to the effective anisotropy perpendicular to the film plane according to:

(6) $$M_{\mathrm{eff}}=\frac{2K_{u,\mathrm{eff}}}{\mu_{0}M_{s}}=M_{s}-\frac{2K_{u,\left[001\right]}}{\mu_{0}M_{s}}$$

with the effective perpendicular uniaxial anisotropy constant *K*_u,eff_. The uniaxial term (*K*_u,[001]_) originates from the aforementioned tensile strain-induced tetragonal distortion (cf. Table 1), adding to the effects of the shape anisotropy and, in turn, increasing the effective anisotropy. Table 3 demonstrates that this behavior is the most pronounced in the 10-nm-thick film and decreases with increasing thickness, in agreement with the thickness dependence of the tetragonal distortion along the [001] axis. This behavior is also reflected in the cubic anisotropies *K*_c_ determined from the IP *H* orientation dependence of *H*_res_. Specifically, *K*_c_ decreases with increasing thickness. For the IP *H* orientation dependence, the absence of a uniaxial contribution supports the observation by Pechan et al. [44] that the strain is isotropic in the film plane.

## 4. Summary and Conclusions

In this work, we studied the magnetic and electronic properties of *L*2_1_-ordered Co_2_MnGa thin films, particularly as functions of the film-thickness-dependent structural and morphological properties. We observed a uniform magnetization throughout the entire film thickness and determined the itinerant and localized characters of the Co *d* and Mn *d* valence electrons and accompanying magnetic moments, respectively. Further, by comparing the Mn 2*p* spectra of Co_2_MnGa with those of Co_2_MnSi [49], a very high degree of spin polarization could be inferred. The combination of high-spin polarization with low Gilbert damping makes the *L*2_1_-Co_2_MnGa films very interesting for potential spin–orbit–torque and spin–transfer–torque devices [68]. In terms of thickness dependence, we made three main observations. First, as the film thickness decreases, a tensile strain, related to a lattice mismatch between the film and substrate, leads to tetragonal distortion of the Co_2_MnGa lattice. Similar tetragonal distortion has been observed in other cubic films, using buffer layer and adding small amounts of a third element [69,70]. This tetragonal distortion likely affects the spin polarization [66] and, in turn, leads to the reduction of the Gilbert damping parameter α (from 2.1 × 10^−3^ to 1.1 × 10^−3^) in thinner films. This demonstrates the possibility of further reducing the Gilbert damping parameter via the film thickness or lattice matched film growth. Second, the crystalline quality increases with increasing thickness [34], resulting in decreased inhomogeneous linewidth broadening (from 14.4 to 5.8 mT). This observation emphasizes the importance of controlling the thickness-dependent film quality in the development of potential materials for device-based applications. Third, the increasing tetragonal lattice distortion with decreasing thickness also results in decreasing uniaxial anisotropy (from −59 to −95 kA/m^3^) perpendicular to the film plane and increasing cubic anisotropy (from 1.9 to 7 kA/m^3^) in the film plane. These features demonstrate that not only the substrate [44] but also the film thickness enables the tuning of the magnetic anisotropies of Co_2_MnGa films, in particular at the thin film limit. Lastly, a potential perpendicular standing spin wave mode was observed in the 80-nm-thick film and enabled quantification of the exchange stiffness (*A* = 16.8 pJ/m). The ability to manipulate the intrinsic magnetic properties with strain-induced epitaxial engineering represents an important springboard for exploiting thin films of Co_2_MnGa in topological spintronic applications. This work enriches the knowledge on the electronic and magnetic properties of Co_2_MnGa films, which are promising in the development of magnetic storage and non-volatile memory technology.

## Figures and Tables

**Table 1:** Structural parameters of Co_2_MnGa films with different thicknesses *t*. Lattice parameters perpendicular to the film plane *c* and in the plane *a* with an error of ±0.001 Å. Misfit strain *f*, root-mean-square (RMS) roughness *S*_q_ and mean lateral surface diameter *D* of the grains.

*t* (nm)	*c* (Å)	*a* (Å)	*f* (%)	*S*_q_ (Å)	*D* (nm)
10	5.727	5.810	3.62	3.08	45 (±8)
20	5.740	5.804	2.83	2.56	56 (±6)
40	5.743	5.792	2.61	3.19	78 (±4)
80	5.751	5.781	2.51	5.44	93 (±5)

**Table 2:** Magnetic parameters of the Co_2_MnGa films extracted from linear fits to the resonance fields and linewidths (Figure 6), including saturation magnetizations from the hysteresis curves (cf. Figure 5).

*t* (nm)	*M*_s_ (kA/m)	*M*_eff_ (kA/m)	*g*	*α* (×10^−3^)	*µ*_0_Δ*H* (mT)
10	857	941	1.97	1.1 ± 0.8	14.4 ± 1.6
20	744	908	1.96	0.8 ± 0.2	14.4 ± 0.5
40	752	884	1.97	1.4 ± 0.1	6.4 ± 0.2
80	760	895	1.95	2.1 ± 0.2	5.8 ± 0.4

**Table 3:** Anisotropy constants of the Co_2_MnGa films determined from simulations of the magnetic field orientation-dependent resonance fields (Figure 7).

*t* (nm)	*K*_u,eff_ (kJ/m^3^)	*K*_u,[001]_ (kJ/m^3^)	*K*_c_ (kJ/m^3^)
10	556	−95	7.0
20	415	−67	3.4
40	430	−75	3.7
80	421	−59	1.9

## Data Availability

The data presented in this study are available on request from the corresponding author.
